# Vestibular paroxysmia associated with congenital vascular malformation: A case report

**DOI:** 10.3389/fnins.2023.1081081

**Published:** 2023-03-06

**Authors:** Fei Liu, Zheng Zhang, Wan Wei, Lin Jiang

**Affiliations:** ^1^Department of Neurology, Affiliated Hangzhou First People's Hospital, Zhejiang University School of Medicine, Hangzhou, Zhejiang, China; ^2^The Fourth School of Clinical Medicine, Zhejiang Chinese Medical University, Hangzhou, Zhejiang, China

**Keywords:** vestibular paroxysmia, congenital vascular malformation, anterior inferior cerebellar artery, vertebral artery, case

## Abstract

Vestibular paroxysmia (VP) is an uncommon paroxysmal disease, characterized by vertigo, tinnitus, and postural unsteadiness. The main reason of VP is neurovascular cross compression, while few cases of VP accompanied with congenital vascular malformation were reported. Here, we describe a 22-year-old patient with VP caused by congenital anterior inferior cerebellar artery (AICA) malformation who completely recovered after taking oral medicine. This report shows that VP caused by congenital vascular malformation can occur in adults and that oral medication is effective.

## Introduction

Vestibular paroxysmia (VP) is a rare disease (<1/2,000) characterized by spontaneous vertigo lasting less than a minute, which responds robustly to oxcarbazepine or carbamazepine. Some patients also have tinnitus, hearing impairment, postural instability, and nystagmus. The prevalence of these symptoms is unknown, as only studies with small sample sizes have been published (Strupp et al., 2016). The underlying pathophysiology of VP is neurovascular cross-compression of the eighth cranial nerve (Shi et al., 2022). Here, we describe an adult patient with VP caused by congenital anterior inferior cerebellar artery (AICA) malformation, as confirmed by digital subtraction angiography (DSA).

## Case report

**Figure 1:**
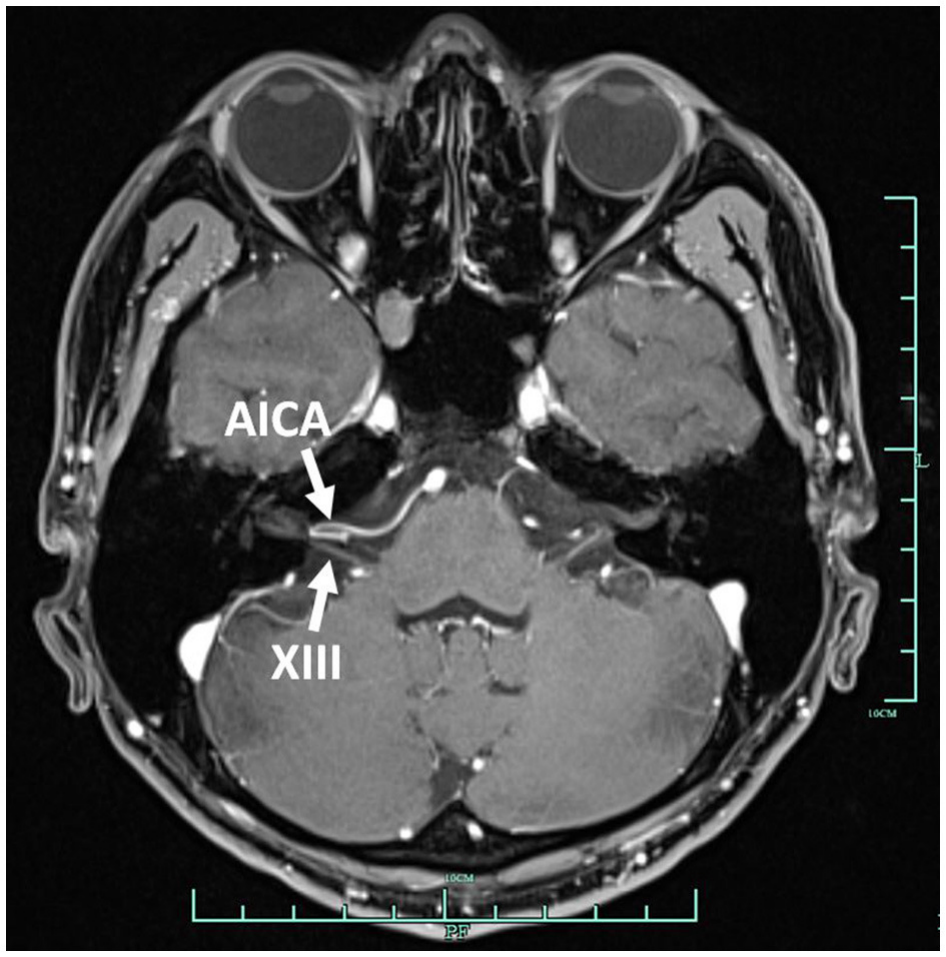
Contrast enhancement MRI of the internal auditory canal. The right AICA compressed the vestibulocochlear nerve. AICA, anterior inferior cerebellar artery; XIII, vestibulocochlear nerve.

**Figure 2:**
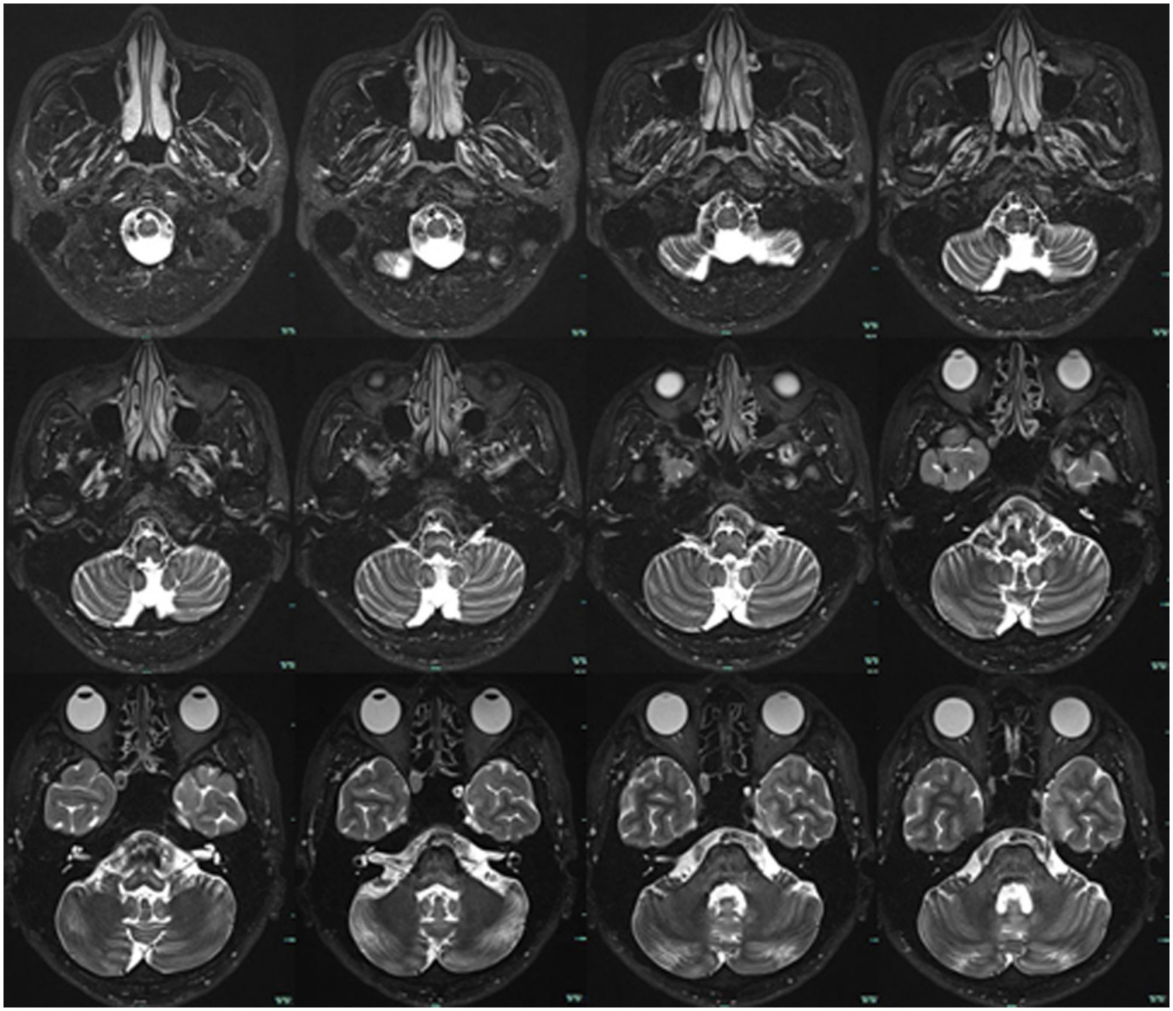
Brain MRI. T2-weighted sequences showed no tumor or other lesions in the cerebello-pontine angle and brainstem.

**Figure 3:**
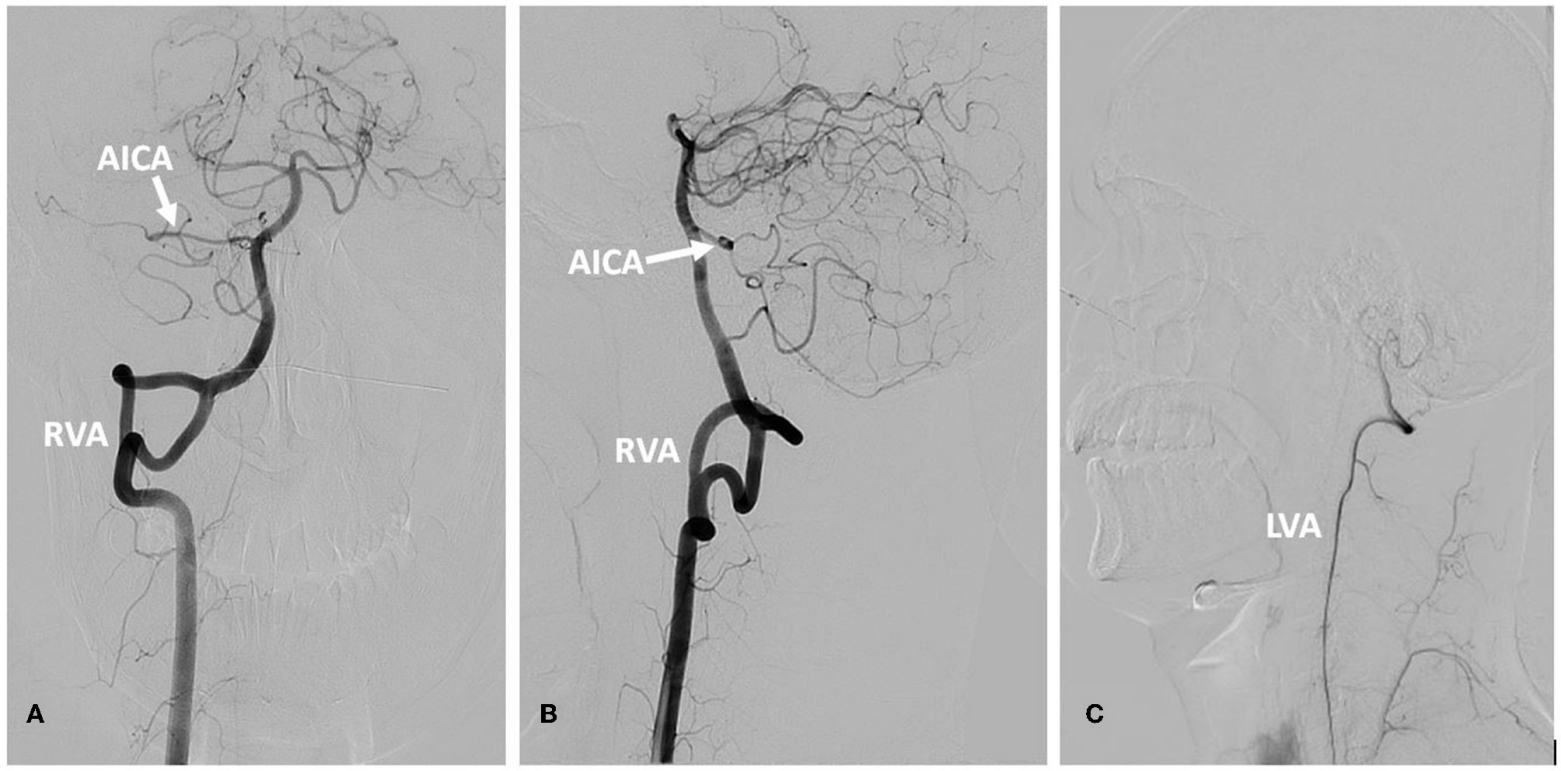
DSA of the vertebral artery and basilar artery. **(A)** Frontal position of the RVA. **(B)** Lateral position of the RVA. **(C)** The LVA. The right vertebral artery was fenestrated, the right AICA was thick, and the left AICA was not visualized **(A, B)**. The LVA terminated in the PICA **(C)**. AICA, anterior inferior cerebellar artery; RVA, right vertebral artery; LVA, left vertebral artery.

In September 2022, a 22-year-old male came to our hospital. He has suffered from paroxysmal non-spinning vertigo for 4 years, with each attack lasting seconds and occurring ~4 times per day; the frequency of the attacks has increased over the last 2 years to ~10 times per day, which has made him very upset. The attacks are not related to head position or hyperventilation and there is an absence of nausea, vomiting, hearing loss, tinnitus, or diplopia, etc. He has no other remarkable medical history. The neurologic examination showed no obvious signs. The binaural hearing, Weber and Rinne tests, and eye movement were normal. The pure tone audiometry and electronystagmography included spontaneous nystagmus, and the gaze test, saccade test, pursuit test, positional test, roll test, and Dix–Hallpike test yielded normal results. Additionally, the head impulse test and vestibulo-ocular reflex were normal. The Romberg sign was negative and coordination movement and the gait test were normal. Furthermore, hyperventilation, positional maneuvers, and horizontal head oscillation did not provoke paroxysmal vertigo or nystagmus. Finally, he immediately relieved the symptoms by taking carbamazepine (0.1 g bid). He was completely free of paroxysmal vertigo after taking carbamazepine (0.1 g bid) over a follow-up of 2 months. This patient is consistent with the diagnosis of definite VP according to the criteria proposed by the Bárány Society in 2016 (Table 1) (Strupp et al., 2016). VP due to neurovascular compression syndrome was diagnosed after a magnetic resonance imaging (MRI) scan with contrast enhancement of the brain, which revealed that the right AICA was compressing the cisternal segment of the vestibulocochlear nerve (Figure 1). The MRI did not identify tumors, such as acoustic neuroma and meningioma, in the cerebello-pontine angle or any other brainstem lesions (Figure 2). The DSA showed that the right vertebral artery (VA) was fenestrated and the left VA terminated in the posterior inferior cerebellar artery (PICA). The right AICA was thick, while the left AICA could not be visualized (Figure 3).

**Table 1:** Diagnostic criteria for VP according to the Bárány Society.

**Definite VP (each point needs to be fulfilled)**	**Probable VP (each point needs to be fulfilled)**
A. At least 10 attacks of spontaneous spinning or non-spinning vertigo	A. At least five attacks of spinning or non-spinning vertigo
B. Duration <1 min	B. Duration <5 min
C. Stereotyped phenomenology in a particular patient	C. Spontaneous occurrence or provoked by certain head movements
D. Response to treatment with carbamazepine/oxcarbazepine	D. Stereotyped phenomenology in a particular patient
E. Not better accounted for by another disease	E. Not better accounted for by another disease

VP, vestibular paroxysmia.

## Discussion

VP is characterized by spontaneous brief vertigo, accompanied by hearing loss, tinnitus, or postural and gait unsteadiness (Brandt et al., 2016). Most patients (70%) have no accompanying symptoms (Steinmetz et al., 2022). It has two age peaks: one begins at a young age because of congenital vertebrobasilar vessel anomalies and the other occurs later in life (40–70 years of age) because of stronger pulsations and vessel elongation (Karamitros et al., 2022). In this report, our young patient only showed non-spinning vertigo, accompanied by congenital vascular variation. Thus, we guess that only the vestibular nerve rather than the cochlear nerve was compressed.

The cause of VP is neurovascular conflict of the vestibular nerve (Hüfner et al., 2008). The vestibular nerve has a much longer cisternal segment, ranging from 14.2 to 19.2 mm (Guclu et al., 2012), where the vestibular nerve transitions from central myelin (glial cells) to peripheral myelin (Schwann cells) (Donahue et al., 2017). The transitional zone of the vestibular nerve is much longer than that of the trigeminal (4 mm), facial (2.5 mm), and glossopharyngeal (1.5 mm) nerves. Compression of the vestibular nerve is more likely in the internal auditory canal, but trigeminal and facial nerve are usually proximally to the CPA (Karamitros et al., 2022). Direct pulsatile compression and brief discharges cause the spontaneous clinical symptoms (Chang et al., 2013). Additionally, long-term vascular compression causes the demyelination of the vestibular nerve and results in hyperexcitability (Dahlin et al., 1989). The AICA (75%) is the most common causative vessel, although the PICA (5%), vein (10%), and VA (10%) can also be implicated (Best et al., 2013). We only found three cases of VP patients in which the type of vessel anomaly was described: the tortuous vertebral artery in a 61-year-old patient (Choi and Kim, 2021), vertebrobasilar dolichectasia in a 66-year-old patient (Han et al., 2018), and PICA elongation in a 37-year-old patient (Silva-Hernández et al., 2019) (summarized in Supplementary Table 1). Childhood VP has been reported, and the main cause is neurovascular cross-compression, but there was no detailed description of vascular malformation (Lehnen et al., 2015). In addition, a few cases of VP associated with congenital vessel malformation have been reported, especially in adult onset patients. The causative vessel of our patient was AICA, and DSA also showed the vessel anomalies of bilateral vertebral arteries and AICA, a congenital malformation, which has never been reported. The compressed point of the vestibular nerve was close to the internal auditory canal rather than the CPA in this patient and no other nerves were affected.

Intracranial fenestration is an anatomical variant related to embryogenesis and perhaps caused by hemodynamic forces, which induce wall shear stress. The vertebrobasilar junction and the proximal basilar artery are the most common sites (Styczen et al., 2022). There are two types of fenestrations: one has a very large window and the other has arterial slits within the lumen. Fenestration may result in aneurysm, stroke, and dissection and may be related to persistent trigeminal artery, arteriovenous malformation, and Moya Moya disease (El Otmani et al., 2020). Our patient had true duplications of the right vertebral artery, showing as a large window, accompanied by one thick AICA on the right side. No obvious aneurysm was observed.

Therapeutically, anticonvulsants, such as oxcarbazepine and carbamazepine, which regulate voltage-gated sodium channels and decrease neuronal activity, are the first choice (Hüfner et al., 2008). When oral medication fails, microvascular decompression, a surgical operation to separate the causative vessel from the compressed vestibular nerve, is an effective treatment (Brandt et al., 2016). After taking carbamazepine, VP did not affect our patient.

## Conclusion

We report an adult case of VP characterized by spontaneous vertigo combined with congenital VA and AICA malformation, which was completely relieved after taking a low dose of carbamazepine. We suggest that VP with congenital vascular malformation can also occur in adults, who should completely recover after taking oral medicine.

## Data availability statement

The original contributions presented in the study are included in the article/Supplementary material, further inquiries can be directed to the corresponding author.

## Ethics statement

The studies involving human participants were reviewed and approved by Affiliated Hangzhou First People's Hospital, Zhejiang University School of Medicine. The patients/participants provided their written informed consent to participate in this study. Written informed consent was obtained from the individual(s) for the publication of any potentially identifiable images or data included in this article.

## Author contributions

FL drafted the manuscript. LJ revised the manuscript. All authors contributed to the acquisition and analysis of data. All authors contributed to the article and approved the submitted version.
