# Supplementary material for: Vestibular paroxysmia associated with congenital vascular malformation: A case report
Source: Front Neurosci. 2023 Mar 6;17:1081081. doi: 10.3389/fnins.2023.1081081 (PMC10025296; doi:10.3389/fnins.2023.1081081)
Supplement: Supplementary file 1 [file Table_1.DOCX]

**Supplemental table 1.** Summaries of patients’ demographic and clinical features

|  | **Case 1** | **Case 2** | **Case 3** |
| --- | --- | --- | --- |
| **Age** (years)  **Sex**  **Past medical history**  **Clinical features**  Vertigo  Tinnitus  Hearing loss  Unsteadiness  Nystagmus  **Associated with head position**  **Brain MRI**  **Therapy**  **Outcome** | 61  Male  Hemifacial spasm  Y  Y  N  N  Y  N  VA/BA tortuousness  Oxcarbazepine  (300mg/day)  Control | 66  Female  Trigeminal neuralgia, hypertension  Y  Y  N  N  N  Y  VA elongation and BA dilatation  Carbamazepine  (300mg/day)  Control | 37  Female  Migraine with aura  Y  Y  N  Y  N  N  PICA elongation  Eslicarbazepine  (800 mg/day)  Control |

Y, yes; N, no; VA, vertebral artery; BA, basilar artery; PICA, posterior inferior cerebellar artery.
